# MRI-based 3D-printed surgical guides for breast cancer patients who received neoadjuvant chemotherapy

**DOI:** 10.1038/s41598-019-46798-1

**Published:** 2019-08-19

**Authors:** Beom Seok Ko, Namkug Kim, Jong Won Lee, Hee Jeong Kim, Il-Young Chung, Jisun Kim, Sae Byul Lee, Byung Ho Son, Hak Hee Kim, Joon Beom Seo, Sung-Bae Kim, Gyung-Yub Gong, Guk Bae Kim, Sangwook Lee, Seung Hyun Choi, Sei Hyun Ahn

**Affiliations:** 1 Division of Breast Surgery, Department of Surgery, Asan Medical Center, University of Ulsan College of Medicine, Seoul, Korea; 2 Department of Radiology, Asan Medical Center, University of Ulsan College of Medicine, Seoul, Korea; 3 Department of Oncology, Asan Medical Center, University of Ulsan College of Medicine, Seoul, Korea; 4 Department of Pathology, Asan Medical Center, University of Ulsan College of Medicine, Seoul, Korea; 5 Department of Convergence Medicine, Asan Medical Center, University of Ulsan College of Medicine, Seoul, Korea

**Keywords:** Breast cancer, Surgical oncology

## Abstract

Magnetic resonance imaging (MRI) is the most accurate technique for evaluating residual tumor after neoadjuvant chemotherapy. However, precise determination of the extent of dispersed residual tumor in the breast following treatment remains a difficult task. We hereby introduce three-dimensional (3D)-printed surgical guides for use in breast cancer patients undergoing breast-conserving surgery after receiving neoadjuvant chemotherapy. We prospectively applied the 3D-printed surgical guides on breast cancer patients who underwent partial breast resection after receiving neoadjuvant chemotherapy. Breasts and tumors were modeled in 3D by using pretreatment magnetic resonance images, and surgical guides were created by using a 3D printer to mark the primary tumor. Out of the five patients who participated in the study, all patients had clear resection margins, and two patients experienced complete pathological remission. There were no recurrences during the median follow-up period of 21.9 months. Thus, our newly-developed 3D-printed surgical guides were useful for accurately marking the extent of breast tumor based on pretreatment magnetic resonance images, which is important for designating the extent of surgery needed in patients who have received neoadjuvant chemotherapy.

## Introduction

**Figure 1:**
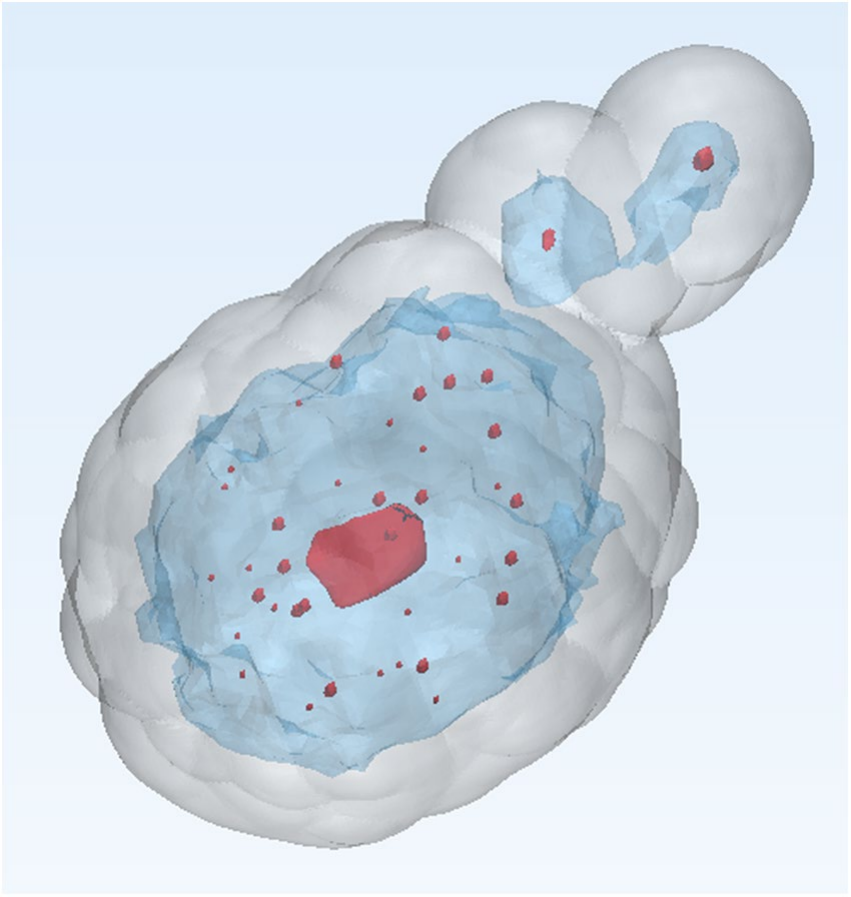
3D image of dispersed tumor cells after neoadjuvant chemotherapy.

Neoadjuvant chemotherapy is helpful for down-staging tumors in locally-advanced breast cancer patients, thus increasing the rate of breast-conserving surgery^1^. Currently, magnetic resonance imaging (MRI) is the most accurate technique for evaluating residual tumor after neoadjuvant chemotherapy^2–4^. However, accurately verifying the extent of tumors based on preoperative magnetic resonance (MR) images is often difficult due to various changes in cancer cells that occur following treatment. In practice, cancer cells are often scattered much wider than the observable remaining lesion (Fig. 1), such that the size of the lesion removed during surgery is greater than the range observed in preoperative images. No current guidance device exists that allows for estimation of an accurate range for breast-conserving surgery after neoadjuvant chemotherapy. We therefore developed three-dimensional (3D) printed surgical guides for breast-conserving surgery in breast cancer patients after neoadjuvant chemotherapy, and evaluated their clinical utility. To the best of our knowledge, this is the first study to apply a surgical guide to breast cancer patients.

## Materials and Methods

**Figure 2:**
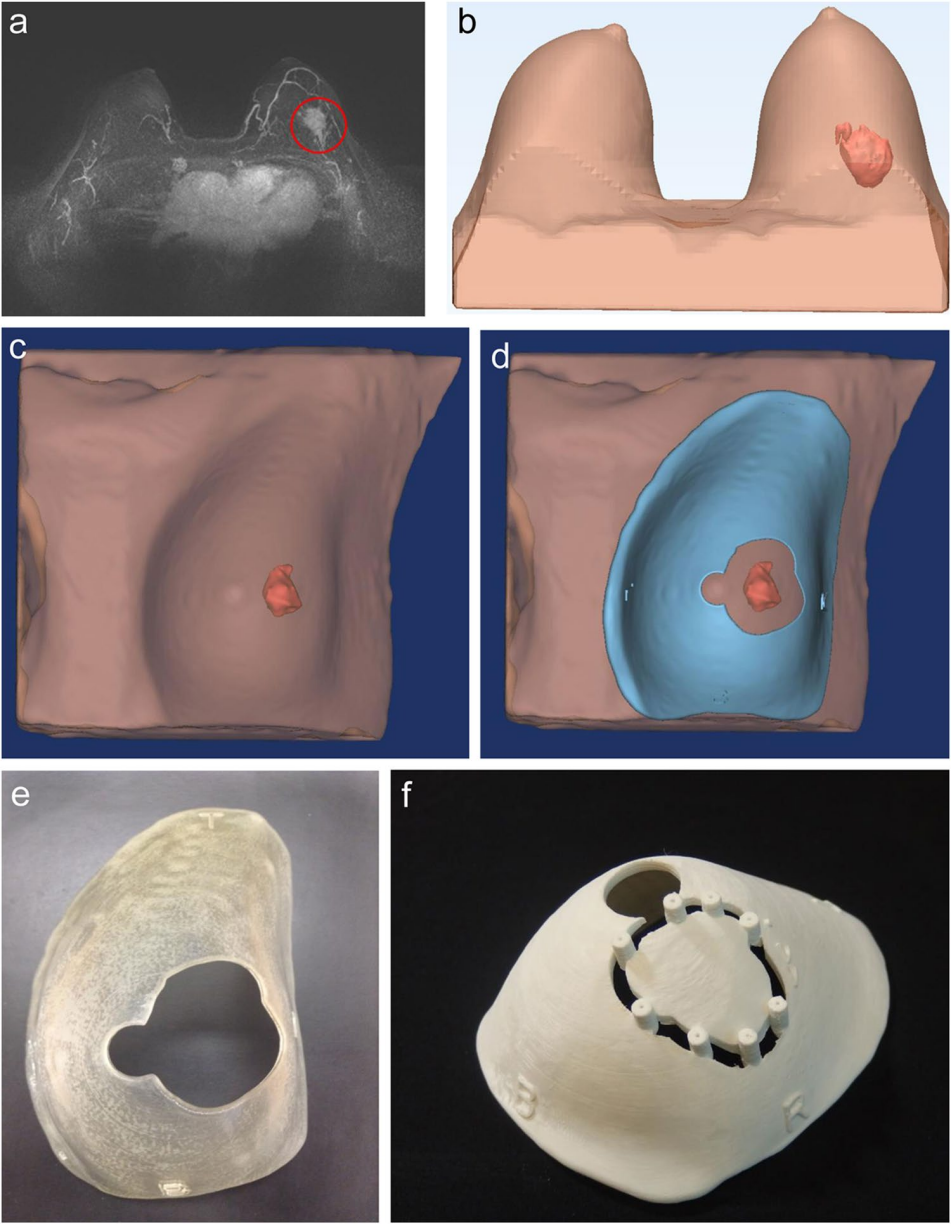
Making of 3D-printed surgical guides based on pretreatment magnetic resonance images. (**a**) Magnetic resonance image before neoadjuvant chemotherapy. (**b**) 3D printer reconstruction of breasts and tumor. (**c**) 3D model of the breast and tumor made prior to neoadjuvant chemotherapy. (**d**) Surgical guide delineating the skin surface and the extent of the tumor. (**e**) Skin-marking type surgical guide. (**f**) Hybrid type surgical guide for skin marking and dye injection.

This prospective pilot study included five breast cancer patients who had received neoadjuvant chemotherapy and were planning to receive breast-conserving surgery. Morphologies of the breasts and tumors were modeled from MR images taken prior to neoadjuvant chemotherapy (Fig. 2a,b).

**Figure 3:**
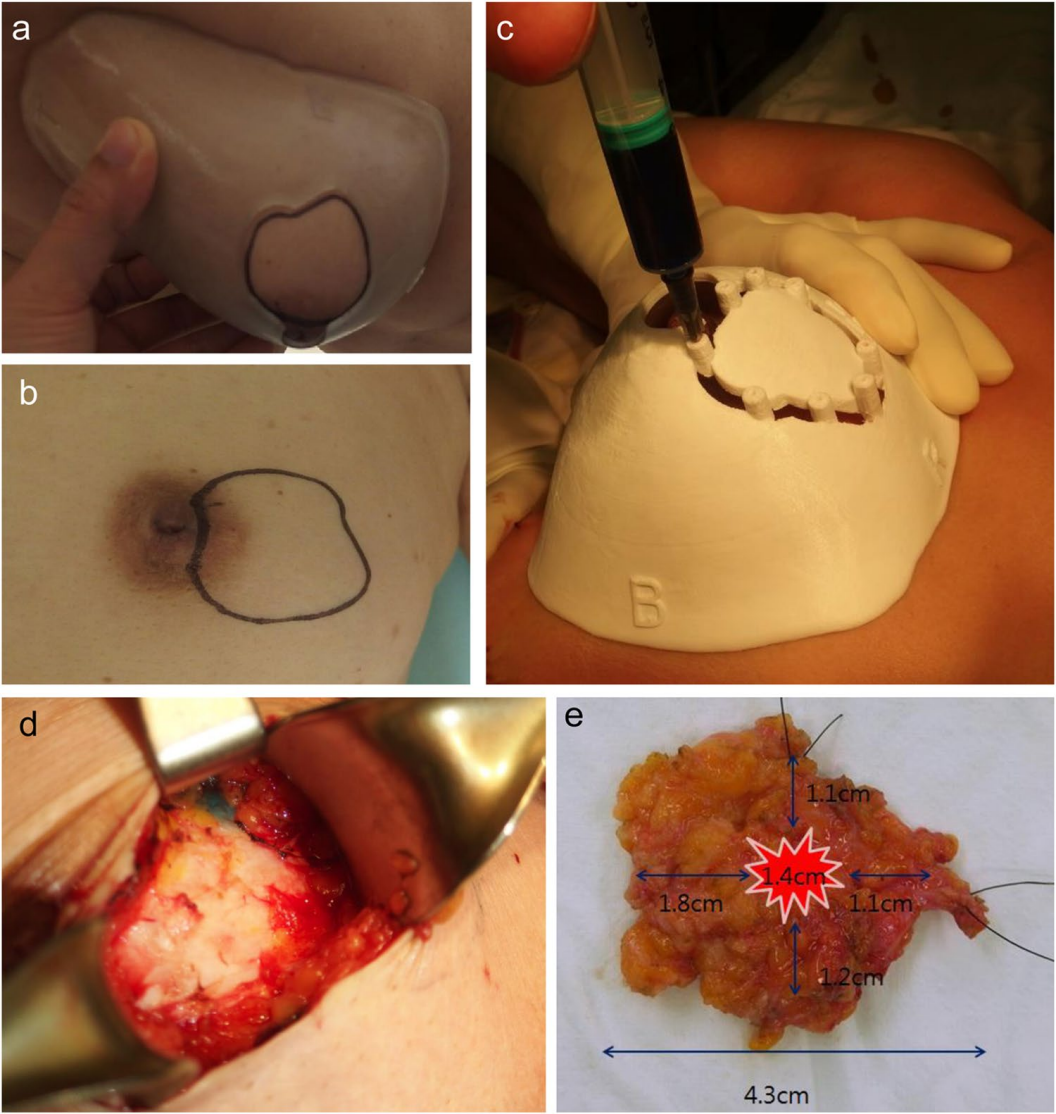
Use of surgical guide for tumor area marking, and confirmation of specimen during/after surgery. (**a**) Using the surgical guide to mark the tumor position with the patient in the supine position. (**b**) Outline of the tumor area on the skin before neoadjuvant chemotherapy. (**c**) Using a hybrid surgical guide for dye injection with the patient in the supine position. (**d**) Blue dye-stained margin used as guide during the surgery. (**e**) Indication of the distance from the surgical guide margin to the resected tumor.

The surgical margins including safety areas (0.5 cm) were designated and projected onto the surgical guiding surface that fits the respective breasts. Morphologies of the breast and nipples were used as landmarks for tailored guidance (Fig. 2c,d). We prepared a surgical guide to accurately target the original tumor area by modeling the tumor and the breast by combining pre-treatment MRI information on the tumor and post-treatment MRI information on the breast. We devised two types of surgical guides—a skin-marking type (Fig. 2e) for drawing guidelines on the skin, and a hybrid type (Fig. 2f) for providing guidelines on the skin and columns for guiding dye injections into tissues. Each had different length needles targeting the exact surgical in-depth margin. The prepared models were saved in stereolithography format and then exported to a 3D printer (Connex3 Objet500; Stratasys Corporation, Rehovot, Israel) for guide creation. The skin-marking type surgical guide was applied to patients in the prone position before general anesthesia to mark the tumor area (Fig. 3a,b). The hybrid type surgical guide was also applied to the skin in the prone position before general anesthesia; after anesthesia, a blue dye was injected in the supine position by using a guide to reduce pain (Fig. 3c). During surgery, tumors were removed based on the skin markings, and injected with the dye (Fig. 3d). Frozen sections were examined to confirm tumor invasion in the resected margins. Distances to the margins from the tumor were measured by a pathologist after surgery (Fig. 3e). The study was approved by the Institutional Review Board of Asan Medical Center (IRB No. 2015-1327) and performed in accordance with the principles of the Declaration of Helsinki. All patients provided voluntary informed consent to participate in the study.

## Results

Five patients (mean age: 48 years) were enrolled from December 2015 to January 2016. The patients were treated with neoadjuvant chemotherapy and targeted therapy according to regional lymph node metastasis status and human epidermal growth factor receptor 2 overexpression status (Table 1).

**Table 1:** Clinical characteristics of the patients.

Number	Age	Multiplicity	Size I	Size II	HG	ER	PR	HER2	SISH amplified	KI-67 (%)	Chemotherapy
1	44	No	2.2	0.6	2	0	0	3		80–90	AC + T + H
2	61	No	3.1	0.3	3	6	0	2	Yes	70–80	AC + T
3	42	No	2.4	1	2	2	0	1		90–100	AC + T
4	49	No	2.5	0.8	2	8	5	0		10–20	AC + T + H
5	48	Yes	7.1	6.8	2	8	8	2	No	20–30	AC + T

Size I: Tumor size determined by using magnetic resonance imaging (MRI) before neoadjuvant chemotherapy.

Size II: Tumor size determined by using MRI after neoadjuvant chemotherapy.

HG: Histological grade.

ER: Estrogen receptor status.

PR: Progesterone receptor status.

HER2: Human epidermal growth factor receptor 2 status.

SHSI: Silver *in situ* hybridization.

AC: Adriamycin and cyclophosphamide.

T: Docetaxel.

H: Herceptin (trastuzumab).

All the patients underwent partial breast resection, and axillary dissection was performed according to axillary lymph node metastasis. Complete pathological remission occurred in two patients. Two patients underwent surgery with ultrasonographic confirmation in the operative field, and the other three patients underwent ultrasonography or mammographic-guided hook-wire (H-wire) localization prior to operation. Sum of the H-wire insertion time and preoperative waiting time was 376 min on average. Median operation time was 78 min (Table 2); the operating time differed depending on whether or not axillary dissection surgery was performed. Frozen biopsy and permanent results showed that the resection margins were clean in all patients. Complete pathological remission occurred in two patients. The median distance from the tumor to the margins was 1.2 cm in the other three patients. No side effects were associated with the application of the surgical guide. There were no recurrences during the median follow-up period of 21.9 months (20.1–22.5 months).

**Table 2:** Preoperative preparation, operation time, and postoperative pathological results.

Number	Surgery	Time I	Time II	Time III	Size	RM 3	RM 6	RM 9	RM 12
1	BCS + ALND	40	280	71	1.4	1.2	1.1	1.8	1.2
2	BCS + SNB	60	404	90	0				
3	BCS + SNB	60	285	78	0				
4	BCS + ALND + SCLND			159	2	2.4	1.5	0.4	0.1
5	BCS + SNB			68	0.7	1.9	2.1	2	2

ALND: Axillary lymph node dissection.

SNB: Sentinel node biopsy.

SCLND: Supraclavicular lymph node dissection.

Time I: H-wire procedure time (minutes).

Time II: Waiting time after H-wire insertion (minutes).

Time III: Operation time (minutes).

Size: Pathological residual tumor size (cm).

RM 3: Resection margin (3 o’clock, cm).

RM 6: Resection margin (6 o’clock, cm).

RM 9: Resection margin (9 o’clock, cm).

RM 12: Resection margin (12 o’clock, cm).

## Discussion

Neoadjuvant chemotherapy has become a standard treatment in advanced breast cancer, and is considered equivalent to adjuvant therapy in terms of survival and overall disease progression^5^. In cases which breast cancer tumors are relatively large, neoadjuvant therapy helps conserve the breast by reducing tumor size. Ensuring negative resection margins and completely removing the tumor is important for successful breast-conserving surgery; for this purpose, accurate measurement of residual tumor size is needed. MRI is the most accurate measure of residual tumor size after neoadjuvant therapy to date^2–4^; however, if small amounts of tumor cells are dispersed following treatment, the extent of the tumor is difficult to be accurately measured. The current operative method is to insert the H-wire in the residual cancer area (as confirmed by breast ultrasonography or mammography), mark the residual cancer area on the skin, and remove it widely around the designated part. However, the insertion of the H-wire is painful, and the procedure takes a considerable amount of time to perform. Adverse events such as hemorrhage or pneumothorax may develop during insertion, and the wire can become severed or lost during surgery^6–9^. In addition, an important disadvantage of H-wire localization is that the wire may penetrate the tumor through the point; therefore, H-wire localization cannot present the entire tumor area, and the area of the tumor on the MRI cannot be marked on the breast.

For a precise and reliable tumor resection, a method for marking the pre-neoadjuvant treatment range of the tumor is necessary. Many attempts have been made to directly mark the MRI-confirmed tumor region onto the breast, but has not been universally applied due to various problems^10,11^. These conventional localization methods have a critical disadvantage in that they cannot present the original area of the tumor prior to treatment.

3D printing is a suitable technology for creating personalized products into desired shapes, and it is used in various medical fields^12,13^. We thus employed 3D printing technology to mark pre-neoadjuvant treatment images directly onto the breast during surgery. By using previously acquired MR images, 3D images were obtained by dividing between the breast and the tumor, and a surgical guide was printed to draw the range of tumors. By using 3D printed surgical guides, we were able to obtain a clean resection margin to completely remove the tumors.

Whereas breast MR images are obtained in the prone position, surgery is carried out in the supine position—therefore, surgical marks drawn on the skin based on MR images would move separately from the tumor during surgery. To solve this problem, we devised special columns for injecting the blue dye, and engineered the length of the column so that the dye would be precisely injected around the tumor. In clinical practice, we found that the blue dye is difficult to inject in the correct position because of the changes in the breast shape caused by posture and gravity. MR images should be obtained in the same position as the position used during the surgery, so that the surgical guide can be made based on supine MR images. Barth *et al*. reported that it was possible to perform a safe and precise operation by applying the 3-D printed form that was produced based on the supine MRI in BCS^14^. In our study, prone MRI-based BSG was useful in successfully removing the tumor; nevertheless, constructing the BSG based on supine MRI may allow for obtaining better clinical results.

In addition, the shape and size of tumors vary according to treatment, so it is important to track the various changes in tumors to distinguish them from normal tissue. We showed that 3D-printed surgical guides may help surgeons accurately mark the extent of the primary tumor based on pretreatment MR images. Quantitative mapping of MR images onto the operative field provides surgeons with additional information prior to neoadjuvant chemotherapy, and can help shorten the operation time. These surgical guides may be useful in breast cancer patients whose tumors are not readily felt by touch or whose tumor range cannot be detected on ultrasonography. In addition, if the H-wire insertion method is replaced with a surgical guide, pain associated with the procedure would be reduced and the procedure time may be shortened. In conclusion, our newly-developed 3D printed surgical guides with quantitative mapping of MRI information were useful in acquiring necessary information about the patients’ tumors prior to neoadjuvant chemotherapy. We expect that this noninvasive method would contribute to reducing the operation time, and to replace conventional procedures such as pre-neoadjuvant chemotherapy clipping or H-wire insertion with ultrasonography.
